# Serum levels of soluble receptor for advanced glycation end products and of S100 proteins are associated with inflammatory, autoantibody, and classical risk markers of joint and vascular damage in rheumatoid arthritis

**DOI:** 10.1186/ar2645

**Published:** 2009-03-11

**Authors:** Yueh-Sheng Chen, Weixing Yan, Carolyn L Geczy, Matthew A Brown, Ranjeny Thomas

**Affiliations:** 1 Diamantina Institute, University of Queensland, Princess Alexandra Hospital, Ipswich Road, Woolloongabba, 4102, Australia; 2 Centre for Infection and Inflammation Research, School of Medical Sciences, University of New South Wales, Sydney, 2052, Australia

## Abstract

**Introduction:**

The receptor for advanced glycation end products (RAGE) is a member of the immunoglobulin superfamily of cell surface receptor molecules. High concentrations of three of its putative proinflammatory ligands, S100A8/A9 complex (calprotectin), S100A8, and S100A12, are found in rheumatoid arthritis (RA) serum and synovial fluid. In contrast, soluble RAGE (sRAGE) may prevent proinflammatory effects by acting as a decoy. This study evaluated the serum levels of S100A9, S100A8, S100A12 and sRAGE in RA patients, to determine their relationship to inflammation and joint and vascular damage.

**Methods:**

Serum sRAGE, S100A9, S100A8 and S100A12 levels from 138 patients with established RA and 44 healthy controls were measured by ELISA and compared by unpaired t test. In RA patients, associations with disease activity and severity variables were analyzed by simple and multiple linear regressions.

**Results:**

Serum S100A9, S100A8 and S100A12 levels were correlated in RA patients. S100A9 levels were associated with body mass index (BMI), and with serum levels of S100A8 and S100A12. S100A8 levels were associated with serum levels of S100A9, presence of anti-citrullinated peptide antibodies (ACPA), and rheumatoid factor (RF). S100A12 levels were associated with presence of ACPA, history of diabetes, and serum S100A9 levels. sRAGE levels were negatively associated with serum levels of C-reactive protein (CRP) and high-density lipoprotein (HDL), history of vasculitis, and the presence of the RAGE 82Ser polymorphism.

**Conclusions:**

sRAGE and S100 proteins were associated not just with RA inflammation and autoantibody production, but also with classical vascular risk factors for end-organ damage. Consistent with its role as a RAGE decoy molecule, sRAGE had the opposite effects to S100 proteins in that S100 proteins were associated with autoantibodies and vascular risk, whereas sRAGE was associated with protection against joint and vascular damage. These data suggest that RAGE activity influences co-development of joint and vascular disease in rheumatoid arthritis patients.

## Introduction

Rheumatoid arthritis (RA) is a chronic inflammatory disease that leads to bone and cartilage destruction and extra-articular complications, including atherosclerotic vascular disease and premature mortality [1]. The receptor for advanced glycation end products (RAGE) has been implicated in the pathogenesis of RA through its ability to amplify inflammatory pathways [2,3]. A member of the immunoglobulin superfamily of cell surface receptors, RAGE binds advanced glycation end products (AGEs), which are non-enzymatically glycated or oxidized proteins, lipids and nucleic acids formed under conditions of oxidative stress and hyperglycemia (reviewed in [4]). In addition to these, RAGE binds some proinflammatory ligands, including members of the S100/calgranulin family, and high mobility group box chromosomal protein 1 (HMGB-1), which is implicated in cell signaling by synergizing with DNA CpG motifs [5,6]. Several RAGE ligands are characteristically overexpressed in RA and psoriatic arthritis, compared to healthy controls [7-9]. S100A8/A9 (calprotectin) and S100A12 (calgranulin C, EN-RAGE) levels are significantly elevated in serum and synovial fluid from RA patients compared to healthy normal donors [3,10]. S100A8/A9 levels are also higher in supernatants of cultured RA synoviocytes than of osteoarthritis synoviocytes [11].

Soluble C-truncated RAGE (sRAGE) lacks the transmembrane and cytosolic domains of the full-length receptor and can prevent proinflammatory effects of RAGE signaling by acting as a decoy [12-14]. For example, in a collagen-induced arthritis (CIA) murine model, treatment with murine sRAGE significantly reduced joint inflammation and destruction [15]. Serum or plasma levels of sRAGE from patients with RA, hypertension or metabolic syndrome were lower than those in healthy subjects [16-18], suggesting that sRAGE levels may identify those RA patients exposed to high levels of RAGE ligands. A gain-of-function Gly82Ser polymorphism in the *RAGE *gene (RAGE 82Ser) occurs more frequently in RA patients than in healthy controls [19]. Monocytes expressing the RAGE 82Ser allele activated a stronger inflammatory response to S100A12 *in vitro *[15]. Although this might be predicted to contribute to enhanced proinflammatory mechanisms in RA, we found no evidence that patients with the RAGE 82S allele had higher levels of inflammation, or a greater likelihood of complicating cardiovascular (CV) events [19].

Most S100 proteins have a mass between 9 and 14 kDa, and are characterized by two calcium binding sites of the EF-hand type (helix-loop-helix) [20]. S100A8 and S100A9, generally functioning as the S100A8/A9 heterocomplex, and S100A12 are implicated in non-infectious chronic inflammatory diseases such as RA, psoriasis and inflammatory bowel disease [21-25]. Longitudinal and cross-sectional studies suggest a relationship between S100A12 and RA disease activity [26-28]. The S100A12 gene is rapidly upregulated in human monocytoid cells and blood monocytes by tumor necrosis factor (TNF) and lipopolysaccharide (LPS), suggesting its production in response to proinflammatory signals in RA [10,25]. S100A12 is a potent monocyte chemoattractant and activates mast cells, which are important effector cells in RA and atherosclerosis [25,29,30]. S100A12 is also proposed to promote proinflammatory activities by binding and activating RAGE [31]. However, these studies were established using a murine model, and since it was later shown that mice have no S100A12 in their genome [20], alternate receptors are implicated [25]. In addition, recombinant S100 ligands may contain contaminating endotoxin, and their effects may not always be fully RAGE dependent [32].

S100A8 and S100A9 regulate leukocyte migration and adhesion [33]. The S100A8/A9 complex has antimicrobial effects, transports arachidonic acid to endothelial cells, and activates expression of endothelial cell adhesion molecules [11,34,35]. Although the receptor for S100A8/A9 complex is still unknown, RAGE has been implicated in some circumstances [36]. Murine S100A8 stimulates proatherogenic activity, such as uptake of low-density lipoprotein (LDL), in macrophages. S100A8 is a key target of oxidation by peroxide, hypochlorite and nitric oxide [37,38]. Furthermore, S100A9 and S100A12 are implicated in vascular damage, whereas sRAGE is associated with vascular protection in atherosclerosis [30,39-41].

The relationship between S100 protein levels and vascular disease or risk factors in RA patients has not been examined to date. We measured serum levels of S100A8, S100A9 heterocomplexes, S100A12 and sRAGE in a previously characterized cohort of established RA patients to identify their possible relationship to joint and vascular damage and risk factors in RA patients [19]. We report associations of each protein with both joint and vascular disease and their risk factors.

## Materials and methods

### Subjects

The cohort of RA patients met the American College of Rheumatology (ACR) 1987 revised criteria for the classification of RA, and has been previously described [42]. These patients presented for a scheduled appointment over a 5-month period (July to November 2003) at our tertiary hospital rheumatology clinic, as described previously [19]. Patients completed a questionnaire detailing CV history, risk factors, treatment, and details of RA. Each patient was clinically evaluated, with chart review to confirm history, at least once. The study protocol was approved by the Princess Alexandra Hospital Research Ethics Committee. Healthy controls (n = 44) without RA or CV disease were recruited by advertisement. All patients and controls signed informed consent to participate. No prospective follow-up was carried out in this study.

### Measurement of S100 proteins

The serum levels of S100A8, S100A9 and S100A12 levels were measured using in-house affinity-purified rabbit polyclonal sandwich ELISAs exactly as described for S100A12 [25]. Antibodies to S100A8 did not cross-react with S100A9 (and did not recognize S100A8/A9 complexes) or S100A12, anti-S100A9 detected free S100A9 and S100A9 as an S100A8/A9 complex; anti-S100A12 was immunoadsorbed with S100A8 and S100A9 [25] and did not cross-react with these when tested by ELISA or immunoblotting. Standard curves were constructed with the relevant recombinant S100 proteins.

### Measurement of sRAGE

sRAGE levels in sera were determined by RAGE Immunoassay (R&D Systems, Minneapolis, MN, USA) in an ELISA format, with wells coated with murine anti-human RAGE mAb in which serum samples (50 μl/well, normally 1:2 v/v dilution) were incubated. A polyclonal capture antibody against the extracellular domain of RAGE was used for detection. The minimum detectable sRAGE concentration is 4.12 pg/ml according to the manufacturer, and the interassay coefficient of variation is < 8% [41].

### Ascertainment of CV events and risk factors, and features of RA

To ascertain CV events, patients were asked for a history, dates and treatments of myocardial infarction, angina, stroke, transient ischemic attack or peripheral vascular disease, and these events were verified by medical record review. Although a number of patients had events prior to the diagnosis of RA, only those CV events that occurred after RA diagnosis were included in the current analysis. Patients with multiple events had only one event counted per person. Myocardial infarction was identified if subjects developed either of; (1) typical rise and fall of biochemical markers (troponin or creatine kinase-MB (CK-MB)) consistent with myocardial necrosis with at least one of the following (a) ischemic symptoms, (b) development of pathological Q waves on the electrocardiogram (ECG), (c) ECG changes indicative of ischemia (ST segment elevation or depression); (2) either new pathological Q waves on serial ECGs or pathological changes of healed or healing infarction [43]. Stroke or transient ischemic attack were identified if subjects had been admitted to the hospital with CT evidence of ischemic occlusion or with carotid endarterectomy, or the subject presented with stroke/transient ischemic attack (TIA) symptoms with significant plaque on the carotid ultrasound and neurological sequelae, with exclusion of subarachnoid hemorrhage and space occupying lesions. Peripheral vascular disease was confirmed if Doppler ultrasonography showed significant large vessel disease.

Cigarette smoking was assessed by questionnaire, which included details about past and present smoking habits, number of cigarettes smoked per day and smoking duration. History of diabetes mellitus was identified if subjects had been diagnosed by a physician, were taking anti-diabetic medications, or had an elevated fasting glucose at the time of the assessment. Family history of CV disease or cerebrovascular attack before age of 65 in first-degree relatives was determined by questionnaire. History was not included if a stroke was deemed hemorrhagic. Body mass index (BMI) was calculated as weight in kilograms divided by the square of the height in meters. Blood pressure was measured at the time of evaluation. History of hypercholesterolemia and hypertension were identified if the diagnoses were recorded in medical records by a physician, if patients were taking lipid-lowering or antihypertensive drugs, or if elevated blood pressure or fasting cholesterol levels were found at the time of the evaluation. The percentage risk of coronary heart disease over the next 10 years was estimated using the 'CVD Risk Calculator' based on the Framingham Study [44] for patients between 30 and 74 years of age and without a history of coronary heart disease. Metabolic syndrome (modified American Heart Association (AHA) standard [45]) was identified by the presence of three or more of these components: (1) BMI > 30; (2) fasting blood triglycerides ≥ 150 mg/dl; (3) blood high-density lipoprotein (HDL) cholesterol (men: < 40 mg/dl (1.03 mmol/l), women: < 50 mg/dl (1.3mmol/l)); (4) blood pressure ≥ 130/85 mmHg; and (5) fasting glucose ≥ 100 mg/dl.

Laboratory data collected at the time of clinical evaluation included fasting total cholesterol, LDL, HDL, very low-density lipoprotein (VLDL), triglycerides, LDL/HDL cholesterol ratio, glucose, creatinine, C-reactive protein (CRP), erythrocyte sedimentation rate (ESR), anti-citrullinated peptide antibodies (ACPA) and rheumatoid factor (RF). A 12-lead ECG carried out within the previous 12 months was scored for evidence of Q waves to ascertain possible silent coronary disease. Creatinine clearance (CrCl) was estimated for each patient on the basis of serum creatinine (SCr), age (years), and ideal body weight (kg) using the Cockcroft and Gault method as follows: CrCl (ml/min) = [(140 - age)(ideal wt)]/833 × SCr (mmol/l) × 0.85 for females [46]. Hand radiographs carried out at the time of evaluation were scored for erosions and joint space narrowing using the modified Sharp score [47].

### Genotyping

High resolution human leukocyte antigen (HLA)-DRB1 genotyping was carried out on buffy coat DNA using PCR and sequence-specific oligonucleotide probes. PCR-based restriction fragment length polymorphism (RFLP) analysis was used to delineate the RAGE Gly82Ser and protein tyrosine phosphatase, non-receptor type 22 (PTPN22) Cys1858Thr polymorphisms as described [15,48]. Shared epitope was considered positive when at least one DRB1 allele was one of the RA susceptibility alleles, as previously described [49].

### Statistical analysis

Data were analyzed using STATA 9.1 (StataCorp, College Station, TX, USA). The variables included age, sex, BMI, current and previous smoking status, RF, ACPA, history of CV events, fasting glucose, homocysteine, cholesterol and triglyceride, ESR, CRP, HDL, LDL, creatinine, CrCl, systolic and diastolic blood pressure, history of diabetes or elevated blood sugar level, history of hyperlipidemia or elevated cholesterol, HLA-DRB1 genotype, Sharp erosion score, Sharp joint space narrowing score, RAGE Gly82Ser polymorphism, history of hypertension or elevated blood pressure, metabolic syndrome (modified AHA standard), serum S100A9, S100A8, S100A12 and sRAGE. Before further analysis, each variable was examined for normal distribution by histogram and box plot. If a variable was not normally distributed, it was transformed (either logarithmic base e or square root transformation) before further analysis. Results are reported as mean ± standard deviation (SD).

Unpaired t tests compared the serum levels of S100A9, S100A8, S100A12 and sRAGE between RA patients and healthy controls. Simple linear regression analysis was used to evaluate the relationship between a variable and the serum concentration of sRAGE or S100 proteins. Variables with *P *< 0.1 using this method were then subjected to multiple linear regression (MLR) analysis. An interaction and residual analysis was also performed on the MLR data. *P *values < 0.05 (two-tailed) were considered statistically significant.

## Results

### Clinical features of the RA cohort

We studied 138 patients with RA (mean age 64.0 years, range 17 to 87 years) and 44 healthy controls (mean age 62 years, range 44 to 80 years) with neither RA nor CV disease. The RA patients were characterized for RA clinical variables, CV risk factors, and RA complications such as vasculitis, radiographic changes, and CV events (Table 1).

**Table 1:** Demographic details, cardiovascular risk factors, features of rheumatoid arthritis (RA) and its control in the study population (n = 138)

**Parameter**	**Value**
Demographics:	
Age (years)	64.0 (10.9)
Females, n (%)	92 (66.7)
Duration of RA (years)	17.6 (13.6)
RF positive, n (%)	83 (61.0)
CV disease:	
History of MI, n (%)	14 (10.1)
History of angina, n (%)	11 (8.0)
History of stroke/TIA, n (%)	9 (6.5)
History of PVD, n (%)	6 (4.4)
Any vascular event, n (%)	26 (18.8)
Risk factors for CV diseases:	
Smoking pack-year history	18 (24)
Current smoker, n (%)	25 (18.1)
History of hypertension, n (%)	47 (34.1)
History of hyperlipidemia, n (%)	33 (23.9)
History of diabetes, n (%)	19 (13.8)
Family history CV disease, n (%)	38 (27.5)
Clinical findings:	
BMI (kg/m^2^)	27.5 (6.9)
Systolic BP (mmHg)	132 (19)
Diastolic BP (mmHg)	78 (10)
Laboratory tests:	
ESR (mm/h)	25 (18)
CRP (mg/l)	13.6 (18.6)
Total cholesterol (mmol/l)	5.3 (1.0)
HDL cholesterol (mmol/l)	1.5 (0.4)
LDL cholesterol (mmol/l)	3.0 (0.9)
TG (mmol/l)	1.6 (1.0)
Homocysteine (μmol/l)	12 (5)
Fasting glucose (mmol/l)	5.6 (1.6)
Serum creatinine (mmol/l)	0.08 (0.05)
CrCl (ml/min)	79.1 (29.9)
Framingham score (%)	11.1 (9.5)
ECG evidence of ischemia, n (%)	2 (1.5)
Severity and feature of RA:	
Radiographic erosion score	24 (35)
Joint space narrowing score	21 (28)
Presence of erosive disease, n (%)	97 (71.3)
History of vasculitis, n (%)	15 (10.9)
Shared epitope, n (%)	103 (75.2)
> 10 mg/day of prednisone, n (%)	9 (6.5)
RAGE polymorphism, n (%)	29 (21.0)

BP, blood pressure; CrCl, creatinine clearance; CRP < C-reactive protein; CV, cardiovascular; ECG, electrocardiogram; ESR, erythrocyte sedimantation rate; HDL, high-density lipoprotein; LDL, low-density lipoprotein; MI, myocardial infarction; PVD, peripheral vascular disease; RAGE, receptor for advanced glycation end products; RF, rheumatoid factor; TG, triglyceride; TIA, transient ischemic attack.

### Increased serum concentrations of the S100 proteins, but not sRAGE, in patients with established RA

**Figure 1:**
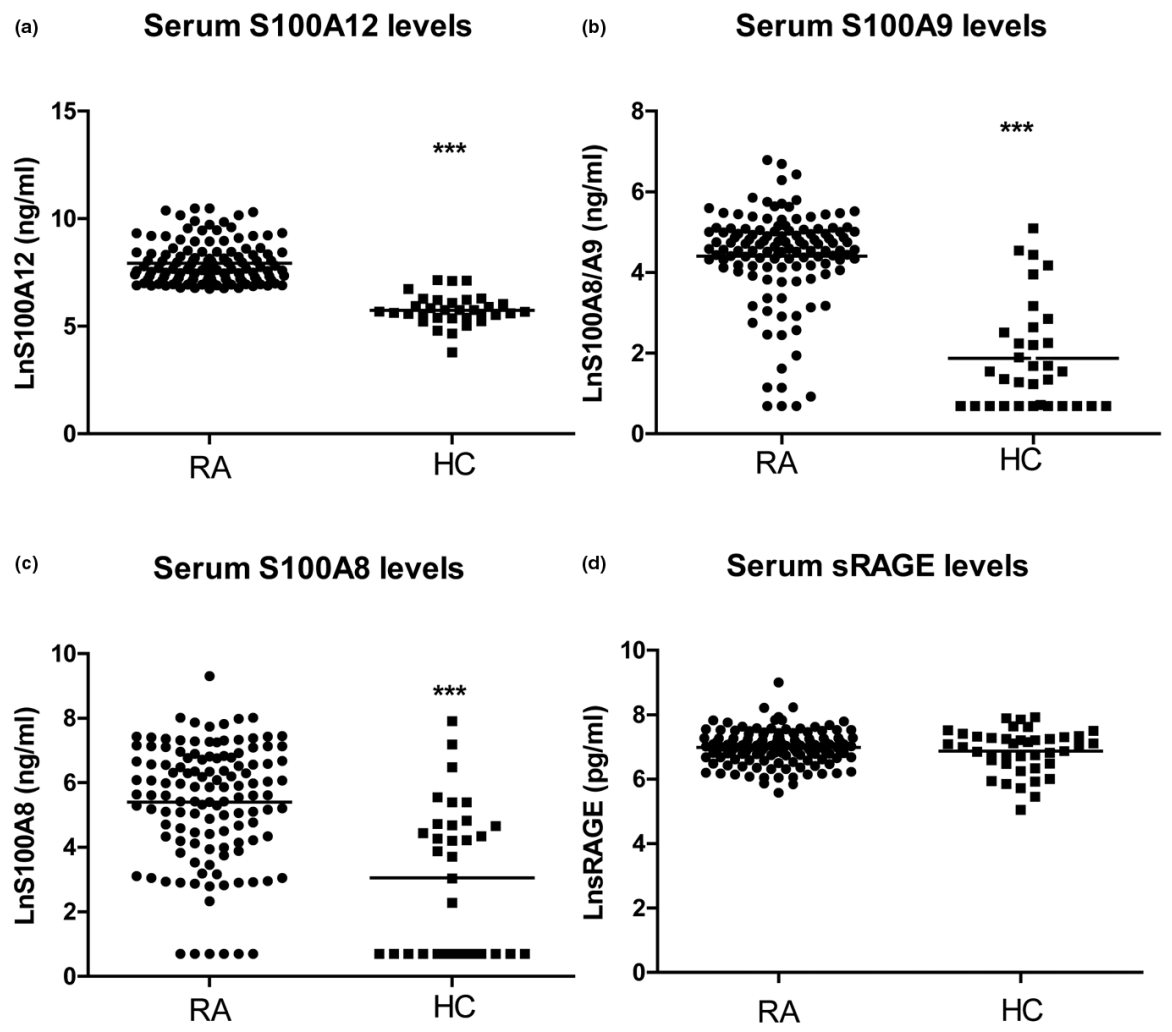
Serum sRAGE, S100A9, S100A8 and S100A12 levels in rheumatoid arthritis (RA) patients and healthy controls. Levels of S100A12 **(a)**, S100A9 **(b)**, S100A8 **(c)**, and soluble receptor for advanced glycation end products (sRAGE) **(d)** were measured in serum of 138 patients with established RA and 44 healthy controls by ELISA. The horizontal line represents the mean value. *** *P *< 0.001, * *P *< 0.05.

Serum levels of S100A9, S100A8 and S100A12 in patients with RA (n = 138) were increased relative to serum levels in healthy controls (n = 44, *P* < 0.001). The S100A9 levels detected in patient sera with an anti-S100A9 antibody that detected S100A9, and S100A9 complexed with S100A8, were some 100-fold lower than those reported in other studies [26,27]. This could reflect differences in the specificity of the anti-calprotectin (an antibody generated against the S100A8/A9 complex) used by others; the anti-S100A9 used by us was generated against pure S100A9. In contrast to the S100 proteins, serum levels of sRAGE were not different (Figure 1a–d).

### Factors associated with serum levels of S100A9, S100A8 and S100A12 in patients with RA

We analyzed the cohort of 138 RA patients for associations between serum levels of S100A9, S100A8, S100A12 and sRAGE with RA clinical variables, CV risk factors, and with complications such as vasculitis, radiographic changes, and CV events. In simple linear regression analysis, we found that serum levels of S100A9 in RA patients were positively associated with the presence of the PTPN22 Cys1858Thr genetic polymorphism, serum levels of S100A12, and serum levels of S100A8 (Table 2, *P* < 0.05). Serum levels of S100A9 in MLR model analysis were positively associated with body mass index, and with serum levels of S100A8 and S100A12 (Table 2, *P* < 0.05).

**Table 2:** Linear regression analysis of S100A9 in 138 rheumatoid arthritis (RA) patients

**Variable**	**β**	**SE**	**t**	***P *value**	**95% Confidence interval**
Univariate analysis:					
ACPA	0.41	0.21	1.91	0.06	-0.01 to 0.83
Body mass index^a^	0.86	0.47	1.84	0.07	-0.07 to 1.78
Diastolic blood pressure	0.02	0.01	1.69	0.09	-0.003 to 0.04
Carriage of PTPN22 Cys1858Thr	0.52	0.24	2.11	0.04	0.03 to 1.00
sRAGE^a^	-0.39	0.20	-1.94	0.06	-0.78 to 0.009
S100A8^a^	0.20	0.06	3.65	< 0.001	0.09 to 0.31
S100A12^a^	0.54	0.10	5.46	< 0.001	0.34 to 0.73
Multivariate analysis:					
Body mass index^a^	0.86	0.41	2.10	0.04	0.05 to 1.67
S100A8^a^	0.11	0.05	2.05	0.04	0.003 to 0.22
S100A12^a^	0.46	0.10	4.50	< 0.001	0.26 to 0.67

Variables that were not listed in the simple and multiple linear regression models did not achieve *P *< 0.1. Adjusted R^2 ^of the multiple linear regression model = 0.25.

^a^Logarithmic (base e) transformation; S100A9 was logarithmically (base e) transformed.

ACPA, anti-citrullinated peptide antibodies; PTPN22, protein tyrosine phosphatase, non-receptor type 22; sRAGE, soluble receptor for advanced glycation end products.

In simple linear regression analysis, serum levels of S100A8 in RA patients were positively associated with ACPA, RF, carriage of HLA-DRB1*0401, RAGE 82S and of the RA-associated HLA-DR shared epitope [50], serum levels of S100A9, S100A12 and radiographic joint space narrowing. Conversely, serum levels of S100A8 were negatively associated with age and serum levels of sRAGE (Table 3, *P* < 0.05). The serum levels of S100A8 in MLR analysis were positively associated with RF, ACPA and serum levels of S100A9 (Table 3, *P* < 0.05).

**Table 3:** Linear regression analysis of S100A8 in 138 rheumatoid arthritis (RA) patients

**Variable**	**β**	**SE**	**t**	***P *value**	**95% Confidence interval**
Univariate analysis:					
ACPA	2.23	0.27	8.34	< 0.001	1.70 to 2.76
Age	-0.03	0.01	-2.15	0.03	-0.06 to -0.002
RF	2.21	0.26	8.33	< 0.001	1.68 to 2.73
Carriage of HLA-DRB1*0401	1.05	0.32	3.26	0.001	0.41–1.69
Carriage of RAGE 82S	0.83	0.40	2.07	0.04	0.04 to 1.62
Carriage of shared epitope	0.94	0.36	2.63	0.01	0.23 to 1.66
Joint space narrowing^a^	0.27	0.12	2.31	0.02	0.04 to 0.50
S100A9^a^	0.48	0.13	3.65	< 0.001	0.22 to 0.75
S100A12^a^	0.67	0.16	4.23	< 0.001	0.36 to 0.99
sRAGE^a^	-0.77	0.31	-2.51	0.01	-1.37 to -016
Multivariate analysis:					
RF	1.45	0.28	5.12	< 0.001	0.89 to 2.01
ACPA	1.22	0.29	4.24	< 0.001	0.65 to 1.79
S100A9^a^	0.34	0.10	3.42	0.001	0.14 to 0.54

Adjusted R^2 ^of the multiple linear regression model = 0.49.

^a^Logarithmic (base e) transformation.

ACPA, anti-citrullinated peptide antibodies; HLA, human leukocyte antigen; RF, rheumatoid factor; (s)RAGE, (soluble) receptor for advanced glycation end products.

In simple linear regression analysis, the serum levels of S100A12 in RA patients were positively associated with ACPA, RF, history of diabetes, and serum levels of S100A8 and S100A9 (Table 4, *P* < 0.05). The serum levels of S100A12 in RA patients in MLR analysis were positively associated with ACPA, a history of diabetes, and serum levels of S100A9 (Table 4, *P* < 0.05).

**Table 4:** Linear regression analysis of S100A12 in 138 patients with established rheumatoid arthritis (RA)

**Variable**	**β**	**SE**	**t**	***P *value**	**95% Confidence interval**
Univariate analysis:					
ACPA	0.75	0.16	4.59	< 0.001	0.43 to 1.08
RF	0.62	0.17	3.66	< 0.001	0.29 to 0.96
Carriage of HLA-DRB1*0401	0.32	0.18	1.79	0.08	-0.03 to 0.66
Carriage of shared epitope	0.32	0.19	1.68	0.10	-0.06 to 0.71
History of diabetes	0.52	0.23	2.23	0.03	0.06 to 0.97
S100A9^a^	0.36	0.07	5.46	< 0.001	0.23 to 0.49
S100A8^a^	0.19	0.04	4.23	< 0.001	0.10 to 0.28
sRAGE^a^	-0.28	0.16	-1.70	0.09	-0.61 to 0.05
Multivariate analysis:					
ACPA	0.63	0.15	4.21	< 0.001	0.33 to 0.93
S100A9^a^	0.31	0.06	4.87	< 0.001	0.18 to 0.43
History of diabetes	0.43	0.20	2.18	0.03	0.04 to 0.82

Variables that were not listed in the simple linear regression model did not achieve *P *< 0.1. Adjusted R^2 ^of the multiple linear regression model = 0.30.

^a^Logarithmic (base e) transformation. S100A12 was logarithmically (base e) transformed.

ACPA, anti-citrullinated peptide antibodies; HLA, human leukocyte antigen; RF, rheumatoid factor; (s)RAGE, (soluble) receptor for advanced glycation end products.

### Factors associated with serum levels of sRAGE in patients with established RA

Simple linear regression analysis showed that serum sRAGE levels in RA patients were negatively associated with current smoking, family history of CV disease, history of vasculitis, diastolic blood pressure, RF, carriage of RAGE 82Ser, and serum levels of CRP and S100A8 (Table 5, *P* < 0.05). MLR analysis of sRAGE levels confirmed the negative associations with RAGE 82Ser, history of vasculitis, and with serum levels of CRP and HDL (Table 5, *P* < 0.05).

**Table 5:** Linear regression analysis of sRAGE levels among 138 patients with established rheumatoid arthritis (RA)

**Variable**	**β**	**SE**	**t**	***P *value**	**95% Confidence interval**
Univariate analysis:					
RF	-0.23	0.09	-2.56	0.01	-0.41 to -0.05
Current smoker	-0.24	0.11	-2.14	0.03	-0.47 to -0.02
Serum creatinine^a^	0.23	0.13	1.77	0.08	-0.03 to 0.49
History of vasculitis	-0.29	0.14	-2.07	0.04	-0.57 to -0.01
Family history CVD	-0.20	0.10	-2.03	0.04	-0.39 to -0.005
HDL^a^	-0.30	0.17	-1.77	0.08	-0.64 to 0.04
CRP^a^	-0.13	0.05	-2.59	0.01	-0.23 to -0.03
Diastolic blood pressure	-0.01	0.004	-2.73	0.007	-0.02 to -0.003
Carriage of RAGE 82S	-0.32	0.10	-3.06	0.003	-0.53 to -0.11
S100A8^a^	-0.06	0.03	-2.51	0.01	-0.11 to -0.01
S100A9^a^	-0.08	0.04	-1.94	0.06	-0.15 to 0.002
S100A12^a^	-0.08	0.05	-1.70	0.09	-0.18 to 0.01
Multivariate analysis:					
Carriage of RAGE 82S	-0.33	0.12	-2.88	0.005	-0.56 to -0.10
HDL^a^	-0.35	0.16	-2.13	0.04	-0.68 to -0.02
CRP^a^	-0.14	0.05	-2.63	0.01	-0.24 to -0.03
History of vasculitis	-0.41	0.15	-2.68	0.009	-0.71 to -0.10

Variables that were not listed in the simple linear regression model did not achieve *P *< 0.1. The selected covariates from simple linear regression (*P *< 0.1) of current smoker, RF, family history of CVD, diastolic blood pressure, creatinine, S100A8 and S100A12 levels were removed from the multiple linear regression model because these covariates did not independently correlate with the dependent variable. R^2 ^of the multiple linear regression model = 0.24. Adjusted R^2 ^= 0.1

^a^Logarithmic base e transformation; sRAGE was logarithmically (base e) transformed.

CRP, C-reactive protein; CVD, cardiovascular disease; HDL, high-density lipoprotein; RAGE, receptor for advanced glycation end products; RF, rheumatoid factor.

## Discussion

We found associations of sRAGE and S100 proteins with clinical inflammatory factors, complications, and CV risk factors in established RA patients. S100 A8, A9 and A12 were all elevated in serum from patients with established RA relative to healthy controls, and their levels were correlated in RA patients. By contrast, serum sRAGE levels did not differ in healthy controls and patients with established RA on treatment. Although a previous study reported reduced levels of sRAGE in RA compared to healthy control sera [17], it seems likely that we observed similar levels because of good control of inflammation in the RA group. In support of this, elevated serum sRAGE levels were generally associated with a more favorable vascular risk profile in our RA cohort, and potentially associated with concomitant reduction in proinflammatory and/or pro-atherogenic RAGE ligand binding.

Despite the generally negative association of sRAGE with vascular risk factors, the single factor that showed a reverse trend in multivariate models was serum HDL. Serum HDL levels were also negatively associated with serum sRAGE among diabetic subjects with CV disease [51]. In contrast, no association with serum HDL and sRAGE was found in patients with essential hypertension [16]. In spite of its known protective role, HDL can become proinflammatory [52-54], and inflammatory HDL may increase the risk of atherosclerosis in SLE and RA patients [55,56]. Moreover, HDL function, which is partly independent of HDL concentration, may be a more critical determinant of the atheroprotective capacity of HDL [57].

The positive association of S100A9 with S100A8 and S100A12 suggests that these proteins may be co-regulated in RA. This is supported by a previous study of S100 proteins in RA patients [58], and is in keeping with the high S100 gene expression profiles reported in RA [59]. Despite the positive associations between of S100A8, A9 and A12 levels, only S100A12 and S100A8 were associated with RA autoantibodies including ACPA and RF. Presence of ACPA or RF predicts a more aggressive RA disease course, including joint erosion and destruction [60]. Although S100A8/A9 from macrophages in RA patients amplified proinflammatory cytokine production in one study [11], the properties of serum S100A8/A9 are still debated. S100A8/A9 expression is seen in macrophages at the cartilage-pannus junction in RA, and expression of S100A8 significantly increased in macrophages in RA patients treated with high dose glucocorticoids compared to pre-treatment samples [61,62]. Interestingly, glucocorticoids amplify LPS-induced S100A8 transcription in macrophages in an interleukin 10-dependent manner. Since we found S100A9 levels were associated with body mass index, it will be of interest to explore the relationship of this protein with endogenous or exogenous glucocorticoids. Recently, S100A9 or S100A8/A9 were reported to promote de-differentiation of dendritic cells and macrophages to myeloid suppressor cells in a tumor-bearing mouse model, suggesting anti-inflammatory effects of S100A9 which may reduce antigen-specific priming, for example, of cytotoxic T cell responses [63]. In support of an anti-inflammatory role for S100A9, S100A8 induced TNF in murine bone marrow cells through TLR4 signaling, and S100A9 negated this activity [64]. We found S100A9 to be associated with dystrophic calcification [39], which may be of relevance to atherosclerotic disease, and warrants future investigation in RA. Thus, the S100A8/A9 complex might have anti-inflammatory properties, or may be related to repair function in damaged or inflamed joints and vessels. It is also plausible that S100A8/A9 has variable effects depending on the presence of other disease factors or treatments. Finally, our assay measured S100A9, whether monomeric or heterocomplexed with S100A8. The ratio of S100A8:A9 may also play a role, given that the heterocomplex can have functions distinct from each protein alone.

In patients with Kawasaki disease, or with chronic hyperglycemia, serum levels of S100A12 were inversely associated with serum levels of sRAGE [65,66]. Although the inverse correlation between sRAGE and S100A12 did not achieve statistical significance in the current study, the associations we found suggest opposing effects on RA severity. S100A12 has potent inflammatory effects. In chronic inflammatory arthritis, S100A12 is expressed by infiltrating granulocytes and by synovial macrophages, is a potent monocyte chemoattractant and activates mast cells to sequester them in inflammatory lesions [25,29].

Our analysis indicates that sRAGE and S100 proteins are associated not only with RA inflammation and autoantibody production, but also with the recruitment of classical vascular risk factors to end-organ damage. This association with vascular risk supports previous reports of low sRAGE and high S100A8/A9 and S100A12 levels in patients with type 1 and type 2 diabetes, and essential hypertension [16,66-68]. These data support evidence from clinical studies of atherosclerosis, suggesting that the roles of classical risk factors and inflammation are difficult to separate in RA [69]. As we observed here, increasing sRAGE levels are associated with a favorable vascular risk profile, potentially associated with concomitant reduction in proinflammatory and/or pro-atherogenic RAGE ligand binding [70,71].

Finally, we observed a novel association of low sRAGE levels with presence of the RAGE 82Ser polymorphism, which is found more frequently in RA patients [15,19]. It is conceivable that this, or other linked polymorphisms in the *RAGE *gene affect splicing of the C-truncated, endogenously secreted form of the receptor, or susceptibility to cell surface RAGE cleavage by matrix metalloproteinases [72], thus altering the ratio of soluble to membrane RAGE.

Several studies have been published which demonstrate the role of sRAGE, S100A8/A9 and S100A12 in the long-term development of vascular disease. These include a negative association between sRAGE levels and coronary artery disease in non-diabetic men [41], prediction of unstable plaque by S100A8/A9 levels in acute coronary syndromes [73], and of accelerated atherosclerosis by high levels of S100A12 in hemodialysis patients [74]. However, this is the first time such an association has been shown with CVD and RA.

## Conclusions

sRAGE and S100 proteins were associated with RA inflammatory factors and autoantibody production, and with the recruitment of classical vascular risk factors to end-organ damage. Consistent with its role as a RAGE decoy molecule, sRAGE had opposing effects to S100A12 and S100A8 in RA. Our data suggest that RAGE may mediate a key pathway coordinating conventional risk factors in the inflammatory RA setting for co-development of joint and vascular disease. Prospective studies will be of interest to determine the utility of these proteins as prognostic biomarkers of joint and vascular damage.

## Abbreviations

ACPA: anti-citrullinated peptide antibodies; ACR: American College of Rheumatology; AGE: advanced glycation end product; BMI: body-mass index; CrCl: creatinine clearance; CRP: C-reactive protein; CT: computed tomography; CV: cardiovascular; ECG: electrocardiogram; ESR: erythrocyte sedimentation rate; HDL: high-density lipoprotein; HMGB1: high mobility group box chromosomal protein; HR: hazard ratio; LDL: low density lipoprotein; MI: myocardial infarction; PCR: polymerase chain reaction; RA: rheumatoid arthritis; RAGE: receptor for advanced glycation end products; RF: rheumatoid factor; SCr: serum creatinine; TG: triglyceride; TIA: transient ischemic attack; TNF: tumor necrosis factor; VLDL: very-low-density lipoprotein.

## Competing interests

The authors declare that they have no competing interests.

## Authors' contributions

YC, CG, MB and RT were involved in conception, design, acquisition, analysis and interpretation of data. WY and CG carried out S100A8, S100A9 and S00A12 assays. YC, CG, MB and RT wrote the manuscript. All authors read and approved the final manuscript.
